# Gait Alterations in Flatfoot Compared to Healthy Controls: A Systematic Review and Meta-Analysis

**DOI:** 10.3390/jcm15093324

**Published:** 2026-04-27

**Authors:** Yoon-Chung Sophie Kim, Albert T. Anastasio, Grayson M. Talaski, Jackson M. Cathey, Sarah C. Ludington, Julia Ralph, Cesar de Cesar Netto

**Affiliations:** 1 Department of Orthopaedic Surgery, Duke University, Durham, NC 27710, USA; albert.anastasio@advocatehealth.org (A.T.A.); grayson.talaski@duke.edu (G.M.T.); jackson.cathey@duke.edu (J.M.C.); sarah.c.ludington@duke.edu (S.C.L.); julia.ralph@duke.edu (J.R.); cesar.netto@duke.edu (C.d.C.N.); 2 Department of Orthopaedic Surgery, St. Vincent’s Hospital, College of Medicine, The Catholic University of Korea, Seoul 06591, Republic of Korea; 3 Department of Orthopaedic Surgery, Wake Forest University School of Medicine, Winston-Salem, NC 27157, USA

**Keywords:** flatfoot, gait analysis, spatio-temporal, kinematics, biomechanics

## Abstract

**Background**: Flatfoot deformity is associated with altered lower extremity biomechanics and functional impairment during gait. However, evidence describing spatio-temporal gait alterations remains heterogeneous and has not been consistently synthesized across studies. **Methods**: A systematic review was conducted in accordance with PRISMA guidelines. MEDLINE (via PubMed) and Scopus were searched through 24 March 2025 for studies evaluating gait characteristics in individuals with flatfoot or progressive collapsing foot deformity. Studies reporting spatio-temporal parameters in both flatfoot and healthy control cohorts were included in quantitative synthesis. Random-effects meta-analyses were performed to evaluate gait velocity, stance duration, stride length, and cadence. **Results**: Fifteen studies met inclusion criteria, of which five provided sufficient data for meta-analysis. Compared with healthy controls, individuals with flatfoot demonstrated longer stance duration and shorter stride length. No differences were observed in gait velocity or cadence. Substantial heterogeneity was present across all pooled outcomes (I^2^ > 80%), reflecting variability in study populations, disease characteristics, and gait analysis methodologies. **Conclusions**: Flatfoot is associated with consistent spatio-temporal gait adaptations characterized by longer stance duration and reduced stride length. Despite heterogeneity among included studies, these findings suggest consistent spatio-temporal gait adaptations that may serve as clinically relevant markers of altered gait mechanics and functional impairment. Further studies with standardized protocols are needed to refine the role of gait analysis in the assessment and management of flatfoot.

## 1. Introduction

Flatfoot deformity, also known as pes planus, represents a common structural condition that alters the biomechanical alignment of the foot and ankle [1]. Although its etiology and severity vary across individuals, flatfoot is frequently associated with impaired dynamic function during gait, which may lead to discomfort and reduced quality of life [2,3]. Advances in musculoskeletal imaging, including radiographs, computed tomography, ultrasonography, and magnetic resonance imaging have improved the understanding of the pathoanatomy of flatfoot deformity. More recently, weight-bearing computed tomography (WBCT) has further enhanced the assessment of anatomical flatfoot deformity under physiological load. In parallel, the concept of progressive collapsing foot deformity (PCFD) has been introduced as a modern nomenclature to describe the progressive and multi-planar nature of flatfoot [4]. Nevertheless, the functional implications of these structural abnormalities have not been fully elucidated.

Quantitative gait analysis offers a dynamic evaluation of lower extremity function and has been increasingly used to assess foot and ankle pathologies [5]. Yet, despite its growing use, there remains a lack of consolidated evidence describing how flatfoot deformity affects spatio-temporal gait characteristics. Previous studies vary widely in methodology, patient populations, and measurement techniques, and few have attempted to correlate functional gait alterations with structural imaging findings [6]. Some prior reviews have focused on treatment outcomes or radiographic progression [7,8]. A recent systematic review and meta-analysis summarized spatio-temporal and kinematic gait changes in flexible flatfoot [9]. However, that review focused specifically on flexible flatfoot and therefore addressed a narrower clinical spectrum than the present study. In contrast, the present review aimed to examine whether common spatio-temporal gait patterns could still be identified across more heterogeneous flatfoot presentations, including PCFD populations.

The present study emphasizes the potential clinical value of spatio-temporal gait parameters as dynamic markers that may provide a conceptual basis for future integration with imaging-based assessment frameworks. We hypothesized that, despite heterogeneity across studies, individuals with flatfoot would demonstrate consistent directional alterations in spatio-temporal gait parameters compared with healthy controls. To address the gap between static structural evaluation and functional assessment, we conducted a systematic review and meta-analysis to synthesize spatio-temporal gait parameters reported in studies comparing individuals with flatfoot and healthy controls. By integrating findings across diverse cohorts, we sought to clarify the characteristic functional gait deviations associated with flatfoot and to provide a foundation for future diagnostic approaches that may integrate dynamic gait analysis with structural imaging modalities, including WBCT.

## 2. Materials and Methods

### 2.1. Study Creation and Initial Search

This systematic review was conducted in accordance with the Preferred Reporting Items for Systematic Reviews and Meta-Analyses Supplementary Material (PRISMA) guidelines [10]. The initial literature search was carried out across two databases—MEDLINE via PubMed and Scopus—encompassing all records retrieved by the search algorithm up to 24 March 2025. The search terms used in this study were (“flatfoot reconstruction” OR “flatfoot” OR “adult acquired flatfoot” OR “flatfoot deformity” OR “progressive collapsing foot deformity” OR “pcfd”) AND (“radiograph” OR “MRI” OR “MR Imaging” OR “magnetic resonance imaging” OR “weight-bearing CT” OR “weightbearing computed tomography” OR “WBCT” OR “imaging”) AND (“gait analysis” OR “motion analysis” OR “kinematics” OR “kinetics” OR “plantar pressure” OR “3D gait” OR “walking”). Imaging-related terms were included in the search strategy to capture studies in which gait findings were evaluated in conjunction with structural characterization of flatfoot, thereby reflecting the broader conceptual framework linking functional assessment with imaging-based evaluation. References were screened for additional articles for potential inclusion. The review protocol was not prospectively registered, which may increase the risk of reporting bias. Ethics approval was not required for this study as it was a systematic review of previously published literature.

### 2.2. Eligibility Criteria

Inclusion criteria were articles that examined patients with PCFD or flatfoot utilizing gait analysis as a primary or secondary analysis method. Further, observational studies, randomized controlled trials, non-randomized prospective studies, and articles with full text were included. Exclusion criteria were articles that did not examine patients with PCFD or flatfoot, articles that did not utilize gait-related methodologies, those with no full text, case reports, animal model studies, cadaveric studies, and reviews.

### 2.3. Article Screening Process

Following the search of the two databases using the specified algorithm, all retrieved articles were imported into Covidence (Veritas Health Innovation, Melbourne, Australia) [11]. Duplicate articles were initially removed automatically, and the remaining articles were then screened by title and abstract according to the inclusion and exclusion criteria. The screening process was conducted by two authors (J.M.C., S.C.L.). After the title and abstract screening, the full texts of the remaining articles were reviewed to determine final inclusion. All conflicts were resolved by discussion amongst the author group.

### 2.4. Data Extraction

Data extraction was performed by three investigators (J.M.C., S.C.L., and J.R.), and results were compared between the investigators to verify that no data had been missed. Data from the included studies were extracted, including first author, year of publication, study type, patient sample size, inclusion and exclusion criteria, patient demographics, PCFD or flatfoot related radiographic metrics, spatio-temporal gait parameters, plantar pressures, joint angle range of motion, type of imaging modality, type of gait analysis modality, type of surgical management, follow-up duration, and additional clinical outcomes.

### 2.5. Methodological Quality Assessment

All observational studies were evaluated using the Methodological Index for Non-Randomized Studies (MINORS) [12]. The MINORS scale distinguishes between comparative and non-comparative studies. For comparative studies, the 12-item checklist assessed the following domains: clearly stated aim, inclusion of consecutive patients, prospective collection of data, endpoints appropriate to the aim of the study, unbiased assessment of endpoints, follow-up period appropriate to the study aim, loss to follow-up less than 5%, prospective calculation of study size, adequate control group, contemporary groups, baseline equivalence of groups, and adequate statistical analyses (ideal score: 24). For non-comparative studies, the 8-item checklist was used, covering the first eight domains listed above (ideal score: 16). Each item was scored as “not reported” (0 points), “reported but inadequate” (1 point), or “reported and adequate” (2 points), reflecting the quality of the study. Two authors (Y.-C.S.K. and G.M.T.) independently performed the scoring and resolved discrepancies by discussion. In the absence of an established universal threshold, methodological quality was categorized as “very low” (0–6 points), “low” (7–10 points), “fair” (11–14 points), and “good/excellent” (≥15 points).

### 2.6. Statistical Analysis

Descriptive statistics were reported as totals, ranges, and percentages. Analyses were performed in R version 4.4.1 (R Foundation for Statistical Computing, Vienna, Austria), utilizing the “meta” and “metafor” packages. Pooled meta-analysis was conducted for studies that reported spatio-temporal parameters for cohorts of both healthy controls and flatfoot patients. Although several included studies involved surgically treated cohorts, only studies reporting comparable spatio-temporal parameters in both flatfoot and healthy control groups were eligible for quantitative synthesis. As a result, the meta-analysis predominantly reflects non-surgical or mixed flatfoot populations with comparable baseline group structures. Pooled estimates of mean gait velocity (meters/second: m/s), stance duration (percent: %), stride length (meters: m), and cadence (steps/min) are presented for aggregate control and flatfoot cohorts, accompanied by 95% confidence intervals (CIs). Heterogeneity was measured with the I2 statistic. Random effects models were employed for all analyses [13]. Between-group differences were descriptively summarized using pooled estimates and corresponding 95% confidence intervals. Given limitations in the available data, formal statistical comparisons between groups were not performed, and results should be interpreted with caution. Note that *p*-values presented in the forest plots reflect tests of heterogeneity and do not indicate statistical significance of differences between cohorts.

## 3. Results

### 3.1. Study Selection

**Figure 1:**
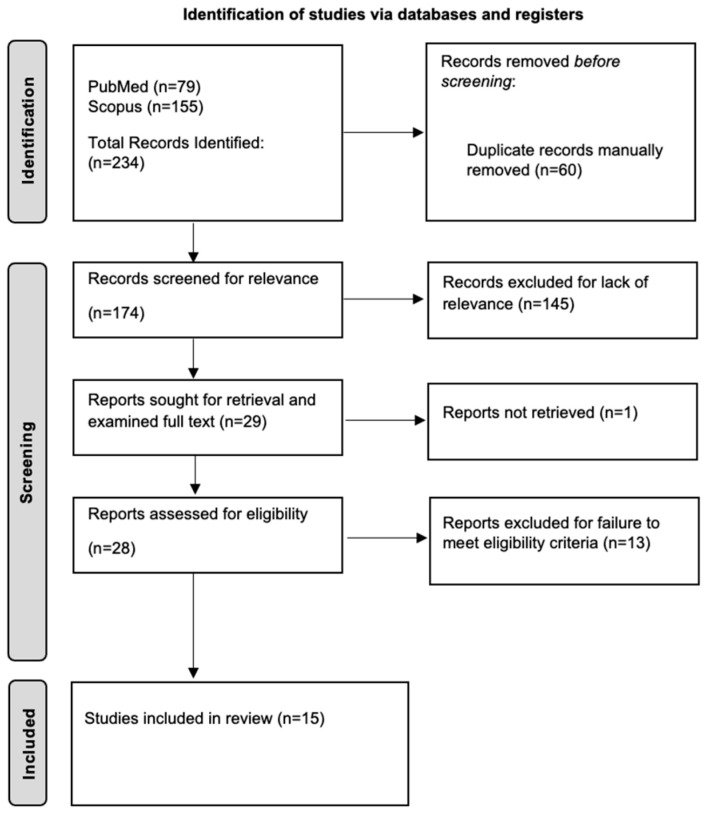
Flow diagram of study selection process.

Our initial search on 24 March 2025 identified 234 studies. Following the removal of duplicates, a total of 174 studies were identified. After screening titles and abstracts, 145 studies were excluded for not meeting the predefined inclusion criteria. Of the remaining studies, one was excluded due to the unavailability of the full text, and 13 were excluded due to reasons including lack of a healthy control group, absence of spatio-temporal gait data, or mismatch in study population. Consequently, 15 studies were included (Figure 1).

### 3.2. Study Characteristics

All included studies were published between 1997 and 2023, with most appearing after 2010. Table 1 describes the characteristics of the included studies. Of the included studies, two [14,15] investigated pediatric populations, seven [7,8,14,15,16,17,18] involved patient cohorts who had undergone flatfoot corrective surgery, and eleven [7,15,18,19,20,21,22,23,24,25,26] utilized healthy individuals as control groups.

### 3.3. Methodological Quality Assessment of the Included Studies

The quality assessment analysis is presented in Table 2. Among the twelve comparative studies, the mean MINORS score was 16.9 (range, 14–22), indicating overall good methodological quality. Nine studies (75%) were rated as “good/excellent” (≥15 points), and three studies (25%) as “fair” (11–14 points), with none classified as “low or very low” quality. Among the three non-comparative studies, the mean MINORS score was 10.0 (range, 8–12), corresponding to low-to-fair methodological quality (one “fair” and two “low”).

### 3.4. Spatio-Temporal Parameters

**Figure 2:**
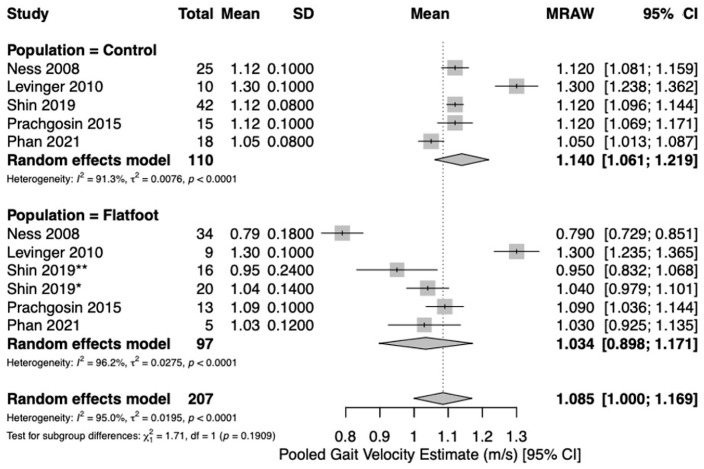
Forest plot to compare gait velocity (meters/second [m/s]) between healthy controls and flatfoot patients [19,21,22,23,24]. The size of each square is proportional to the weight of the corresponding study in the meta-analysis. Horizontal lines represent 95% confidence intervals, and diamonds indicate pooled estimates. * Shin 2019 = moderate flatfoot. ** Shin 2019 = severe flatfoot.

**Figure 3:**
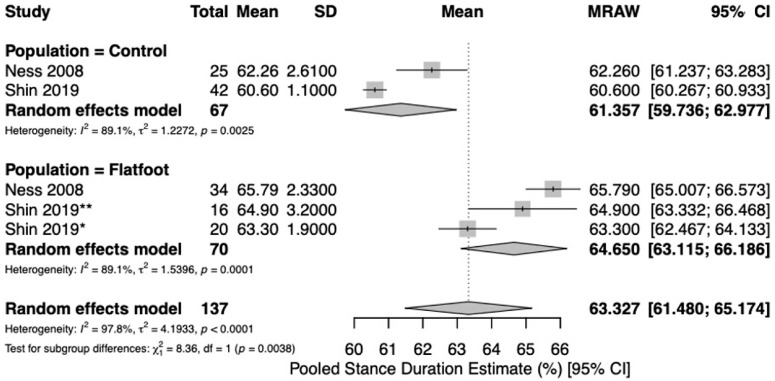
Forest plot to compare stance duration (%) between healthy controls and flatfoot patients [22,24]. The size of each square is proportional to the weight of the corresponding study in the meta-analysis. Horizontal lines represent 95% confidence intervals, and diamonds indicate pooled estimates. * Shin 2019 = moderate flatfoot. ** Shin 2019 = severe flatfoot.

**Figure 4:**
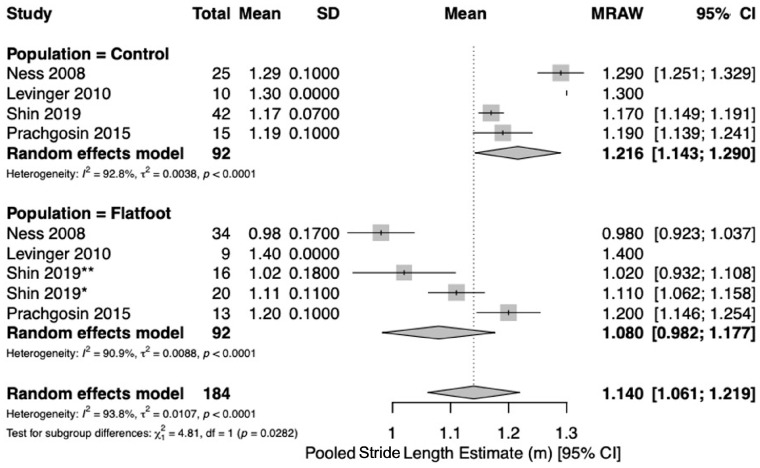
Forest plot to compare stride length (meters [m]) between healthy controls and flatfoot patients [21,22,23,24]. The size of each square is proportional to the weight of the corresponding study in the meta-analysis. Horizontal lines represent 95% confidence intervals, and diamonds indicate pooled estimates. * Shin 2019 = moderate flatfoot. ** Shin 2019 = severe flatfoot.

**Figure 5:**
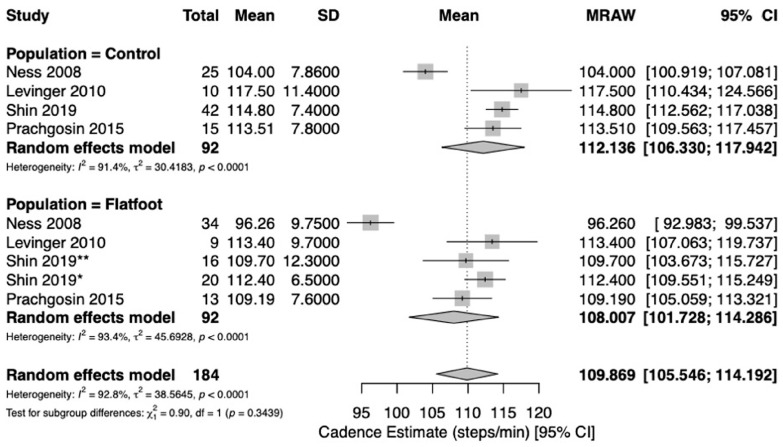
Forest plot to compare cadence (steps/min) between healthy controls and flatfoot patients [21,22,23,24]. The size of each square is proportional to the weight of the corresponding study in the meta-analysis. Horizontal lines represent 95% confidence intervals, and diamonds indicate pooled estimates. * Shin 2019 = moderate flatfoot. ** Shin 2019 = severe flatfoot.

Five studies [19,21,22,23,24] reported spatio-temporal gait parameters as outcome measures for flatfoot and healthy control cohorts. Pooled meta-analysis was conducted for outcomes when at least two studies with comparable design reported measures of central tendency (mean) and variance (standard deviation) for spatio-temporal parameters for healthy controls and flatfoot patients. Five studies [19,21,22,23,24] reported on gait velocity (meters/second [m/s]). Analysis revealed similar estimates of pooled mean gait velocity between flatfoot (1.03 m/s; 95%CI [0.90, 1.17], I^2^ = 96.2%) and healthy control cohorts (1.14 m/s; 95%CI [1.06, 1.22], I^2^ = 91.3%) (Figure 2). Two studies [22,24] reported on stance duration (%). The pooled estimate of percent stance duration was higher among flatfoot patients (64.7%; 95%CI [63.1, 66.2], I^2^ = 89.1%) compared to healthy controls (61.4%; 95%CI [59.8, 63.0], I^2^ = 89.1%) (Figure 3). Four studies [21,22,23,24] reported on stride length (meters [m]). The pooled estimate of mean stride length in flatfoot patients (1.08 m; 95%CI [0.98, 1.18], I^2^ = 90.9%) was lower than that of healthy controls (1.22 m; 95%CI [1.14, 1.29], I^2^ = 92.8%) (Figure 4). Four studies [21,22,23,24] reported on cadence (steps/min). Pooled mean cadence estimates were similar for flatfoot patients (108 steps/min; 95%CI [102, 114], I^2^ = 93.4%) and healthy controls (112 steps/min; 95%CI [106, 118], I^2^ = 91.4%) (Figure 5).

### 3.5. Results of Individual Studies

Shin et al. [24] found that flatfoot patients demonstrated significantly reduced cadence, gait velocity, stride length, and step width compared to healthy controls. They presented kinematic characteristics of inter-segmental foot motion during barefoot gait at a comfortable speed in adult acquired flatfoot patients using a multisegmental foot model (MFM) with a 15-marker set (DuPont Foot Model) [25,26]. Severe flatfoot (Meary angle > 20°) was associated with decreased range of motion (ROM) in the sagittal and transverse planes of the hindfoot, transverse plane of the forefoot, and sagittal plane of the hallux. Loss of hindfoot adduction during terminal stance and pre-swing, along with a more supinated and abducted forefoot position throughout the gait cycle, were observed in severe cases. Plantarflexion of the hindfoot in pre-swing decreased in proportion to deformity severity. The authors suggest a possible threshold (Meary angle < 20°) below which normal foot kinematics may be preserved.

Ness et al. [22] reported that patients with posterior tibial tendon dysfunction (PTTD) had significantly reduced stride length, cadence, and gait velocity, with prolonged stance duration compared to healthy controls. Kinematic analysis using the Milwaukee Foot Model revealed diminished hindfoot dorsiflexion with increased eversion, reduced forefoot plantarflexion with abduction shift and loss of varus thrust, and decreased hallux ROM with diminished dorsiflexion. These findings highlight marked multisegmental gait alterations in PTTD, supporting the need for improved orthotic and bracing strategies, as well as providing a reference framework for evaluating surgical outcomes.

Prachgosin et al. [21] utilized the Oxford Foot Model to assess medial longitudinal arch (MLA) biomechanics and segmental kinematics, reporting that flatfoot participants demonstrated significantly greater peak eversion moments at the MLA and reduced peak MLA deformation angles compared with normal controls. Kinematic analysis revealed increased hindfoot plantarflexion and internal rotation, as well as greater forefoot abduction during specific stance subphases. In terms of ground reaction forces (GRF), the flatfoot group exhibited reduced peak vertical GRF in late stance and increased peak medial GRF in mid stance. These findings indicate higher deforming forces, greater segmental motion, reduced MLA flexibility, and altered loading patterns in flatfoot gait.

Levinger et al. [23] also utilized the Oxford Foot Model and reported that participants with flat-arched feet demonstrated greater peak forefoot plantarflexion, forefoot abduction, and hindfoot internal rotation compared with those with normal arches. They also exhibited reduced peak forefoot adduction and a trend toward increased hindfoot eversion. These altered kinematic patterns suggest increased pronation during gait, potentially elevating the risk of overuse injuries.

Phan et al. [19] utilized CT-derived 3-dimensional (3D) bone models combined with biplanar fluoroscopy (BPF) technique [29,30] to capture in vivo bone-to-bone kinematics during gait, enabling quantification of joint rotations, ROM, and relative articular surface velocities across multiple foot joints. They revealed that flatfoot subjects exhibited significantly higher relative speeds at the tibiotalar, subtalar, and calcaneocuboid articular surfaces compared to normal controls, indicating increased joint instability. Kinematic analysis revealed greater plantarflexion in the tibiotalar joint, and increased eversion and external rotation in the talonavicular joint during stance. Additionally, flatfoot participants showed larger ROM in inversion/eversion and internal/external rotation at the tibiotalar joint. These findings highlight abnormal multi-joint kinematics and instability during mid- to terminal stance in flatfoot gait.

Wang et al. [20] evaluated lower-limb biomechanics in adults with flexible flatfoot during walking. Using a 3D motion capture system with force plates, they found that flatfoot participants exhibited significantly greater ankle eversion angles and moments, as well as increased knee internal rotation during stance compared with controls. Hip abduction moments were also elevated, suggesting compensatory proximal joint mechanics. Spatio-temporal parameters such as gait velocity and step length were reduced in the flatfoot group. These results indicate that flexible flatfoot alters multi-joint kinematics and kinetics beyond the foot, potentially increasing the risk of overuse injuries in the lower limb.

### 3.6. Summary and Consistency of the Evidence

Across the five key studies, flatfoot—whether symptomatic adult acquired flatfoot, PTTD, or radiographically defined flat-arched morphology—was consistently associated with shorter stride length and prolonged stance duration compared with healthy controls. In contrast, pooled analyses did not demonstrate significant differences in gait velocity or cadence between flatfoot and healthy controls. Common kinematic alterations included increased hindfoot eversion and internal rotation, greater forefoot abduction, reduced forefoot adduction, diminished sagittal plane motion at the hindfoot and hallux, and aberrant loading patterns reflected in GRFs. A summary is provided in Table 3.

## 4. Discussion

In this review, flatfoot cohorts consistently demonstrated shorter stride length and prolonged stance duration compared with healthy controls, while cadence and self-selected gait velocity showed no clear differences. This pattern suggests that individuals with flatfoot tend to maintain a near-preferred cadence but adopt a conservative gait strategy characterized by shorter steps and longer ground contact to enhance stability [24,31,32]. As a result, overall gait velocity remains comparable to controls, despite reductions in stride length. The absence of significant velocity differences at the meta-analytic level may also reflect study heterogeneity and differences in testing conditions, such as self-selected versus controlled gait velocities. Given the substantial heterogeneity across outcomes (I^2^ ranging from approximately 89% to 96%), pooled estimates should be interpreted as exploratory rather than confirmatory.

A total of 15 studies were ultimately included in the present meta-analysis, and considerable heterogeneity was observed in study design and patient populations, not only across the overall dataset but also among the remaining studies beyond the five that served as the primary sources for spatio-temporal parameter analysis. Specifically, Colo [16], Oeffinger [14], and Parsons [17] investigated cohorts undergoing flatfoot corrective surgery without a healthy control group, while Chong [15], Schuh [7], and Barske [18] compared surgical cohorts with healthy controls. Ellis [8] focused on patients who had undergone lateral column lengthening, stratifying them according to the presence or absence of lateral foot pain and evaluating plantar pressure profiles. Khan [28] examined only female patients with flatfoot, comparing them with healthy controls, whereas Petje [27] analyzed patients with a history of Lisfranc injury who developed mobile flatfoot, again contrasted with healthy individuals. Taken together, these examples underscore the heterogeneity across the included studies and highlight the relative scarcity of investigations that directly and uniformly addressed the topic of gait alterations in flatfoot compared with healthy controls.

However, through this analysis we were able to identify a set of common findings that may serve as potential dynamic biomarker candidates differentiating flatfoot from healthy controls. In the spatio-temporal domain, flatfoot cohorts tended to exhibit reduced stride length, while gait velocity and cadence were generally comparable to controls at the meta-analytic level. Kinematic alterations were also consistent, with greater hindfoot eversion and forefoot abduction, together with reduced sagittal plane motion of both the hindfoot and the hallux. In terms of functional loading, flatfoot was associated with increased MLA eversion moments, decreased MLA deformation angles, and abnormal GRF patterns. Although the included studies employed heterogeneous models and analytical methods, these parameters showed a consistent direction of change in flatfoot, supporting their potential role as dynamic biomarker candidates. These candidate parameters are summarized in Table 3.

None of the included studies utilized WBCT for structural characterization, limiting the ability to directly relate dynamic gait alterations to precise 3D osseous morphology. Recent work using WBCT has identified peritalar subluxation as a key imaging biomarker distinguishing symptomatic PCFD from asymptomatic flatfoot, with the minimum sinus tarsi distance emerging as the strongest predictor of symptoms, using a threshold of 1.9 mm [33]. Correlating such WBCT-derived parameters with gait-based spatio-temporal and kinematic markers—such as shorter stride length and prolonged stance duration, increased hindfoot eversion, and greater forefoot abduction—may yield clinically actionable thresholds to guide diagnosis, monitoring, and surgical planning for symptomatic PCFD. Given that surgical decision-making is multifactorial and influenced by deformity flexibility, severity, patient age, functional demands, and surgeon expertise [34], our findings may support the development of multimodal assessment strategies that combine dynamic (gait) and static (WBCT) evaluation techniques. This perspective is consistent with the conclusion of Ness et al. [22], who emphasized that gait alterations in flatfoot provide a foundation for evaluating the efficacy of surgical correction and may serve as an objective benchmark for improved treatment planning, including both orthotic and operative interventions.

From a clinical perspective, the observed pattern of shorter stride length and prolonged stance duration may reflect a compensatory gait strategy adopted to enhance stability in the setting of altered foot biomechanics. These adaptations may contribute to increased energy expenditure, reduced walking efficiency, and fatigue during prolonged ambulation, potentially impacting quality of life in patients with flatfoot, particularly those with PCFD. Recognition of these alterations may also inform treatment planning, as orthotic or surgical interventions aimed at restoring alignment and support may help normalize gait patterns. While further validation is needed, spatio-temporal gait parameters may serve as objective functional markers for identifying and monitoring gait abnormalities in flatfoot.

Despite these insights, several limitations of this meta-analysis should be acknowledged. First, the number of studies directly comparing flatfoot patients with healthy controls was relatively small, and substantial heterogeneity was observed across study designs, patient populations, and outcome measures. This variability included differences in age groups, sex distribution, definitions of flatfoot, and gait analysis protocols. Because pooled estimates were calculated separately for flatfoot and control cohorts, study-level pairing was not preserved, limiting direct statistical comparison between groups. Furthermore, assessment of reporting bias and certainty of evidence was not feasible due to the limited number of studies included in each meta-analysis, restricting the reliability of such evaluations. Second, spatio-temporal parameters were not uniformly reported across studies, and essential statistical information, such as standard deviations, was occasionally missing, limiting the ability to pool data for all relevant outcomes. Third, most included studies were cross-sectional in nature, precluding longitudinal evaluation of disease progression or the effects of surgical correction over time. Fourth, no included study incorporated WBCT, limiting the ability to directly correlate dynamic gait alterations with three-dimensional structural parameters of the foot. Finally, the methodological quality of the included studies, as assessed by the MINORS criteria, varied from low to fair in non-comparative studies and from fair to good in comparative studies, indicating a need for more rigorously designed prospective trials. Taken together, these limitations underscore that the present findings should be interpreted with caution and highlight the need for future high-quality, standardized studies to establish robust dynamic biomarkers and their relationship with structural imaging parameters in flatfoot deformity.

## 5. Conclusions

This systematic review and meta-analysis demonstrated that flatfoot patients exhibit shorter stride length and prolonged stance duration compared with healthy controls, while gait velocity and cadence remain largely preserved. These findings highlight the value of spatio-temporal gait parameters as dynamic indicators of functional impairment in flatfoot. Future studies integrating such gait-based measures with advanced imaging modalities may help establish clinically feasible biomarkers within multimodal assessment and surgical decision-making.

## Figures and Tables

**Table 1:** Summary of included studies on gait analysis in flatfoot.

Author (Year)	Overall Population	Patient Cohort	Study Groups	Group Size	Inclusion Criteria	Exclusion Criteria	Imaging Modality	Gait Analysis	Age (Mean (SD) or [Range])	Sex (F, M)	BMI (kg/m^2^; Mean (Range/SD) or Median [IQR])
Colo 2021 [16]	22	AAFD	-	-	AAFD patients with PTT insufficiency (stage II), unresponsive to conservative measures (NSAIDs, orthotics and physical therapy) for at least 6 months who underwent lateral open wedge calcaneus osteotomy with bony allograft augmentation combined with tibialis posterior and tibialis anterior tenodesis	Associated talonavicular arthrodesis, history of diabetes, less than 2-year of follow-up and bilateral surgery	X-ray	Accelerometric analysis (Free4Act^®^ F4A sensors, Bologna, Italy)	58.8 (17.4)	9, 13	-
Oeffinger 2000 [14]	8	Children with PPV	-	-	Symptomatic PPV, had undergone a lateral column lengthening, failed prolonged non-operative therapeutic measures including orthotics	-	X-ray	EMED platform system (4 sensors/cm^2^)	13.6 (3.8)	-	-
* Phan 2021 [19]	23	Flatfoot male	Healthy	18	-	-	X-ray + biplanar fluoroscopy	Non-weight bearing CT with Seg3D	24.2 (1.8)	0, 18	-
			Flatfoot	5					24.4 (0.5)	0, 5	
Wang 2019 [20]	19	AAFD	Healthy	7	Healthy subjects without traumatic or surgical history of the lower limbs and be examined by two experienced foot and ankle surgeons	AAFD pts who refused to take the required radiographs	single-plane fluoroscopy + CT scan	Single fluoroscopy system + FluoMotion software (Innomotion Inc., Shanghai, China)	44.3 (7.3)	3, 4	28.3 (5.8)
			Stage II AAFD	12					42.6 (6.2)	7, 5	29.9 (7.5)
* Prachgosin 2015 [21]	28	Flatfoot	Healthy	15	18–50 yo, BMI < 25, recruited from local population in southern Thailand	any neuromuscular-skeletal diseases were excluded	X-ray + footprint analysis	3D motion analysis lab (MLA deformation angles, hindfoot and forefoot kinematics, GRFs)	32.7 (8.9)	14, 1	21.1 (1.6)
			Flatfoot	13					24.9 (3.3)	10, 3	22.7 (1.9)
Parsons 2010 [17]	32	AAFD	-	-	Patients who failed nonoperative treatment involving physiotherapy and orthoses for a minimum of 3 months, willing to undergo modified Cobb technique	Osteoarthritis in the ankle and midfoot	MRI + US	Single heel rise test + AOFAS score	[44–66]	28, 4	-
Chong 2015 [15]	32	Flatfoot children	Arthroereisis	7	PPV deformity, painful symptom refractory to conservative treatment for at least 6 months, and independent ambulation without assistive device	Patients taking medication affecting motor control, those unable to follow direction or participate in a gait lab study	X-ray	Kinematic motion analysis + pedobarometry	12.8 (8–17)	-	-
			LCL	8							
			Healthy	17					10.2	-	-
* Ness 2008 [22]	59	PTTD	PTTD	34	-	-	X-ray	Vicon Motion Analysis System	52.8 (9.5)	30, 4	32 (7.5)
			Healthy	25					41.3 (12.5)	12, 13	26.3 (3.8)
* Levinger 2010 [23]	19	Flatfoot	Healthy	10	-	One flatfoot subject was excluded due to a technical issue	X-ray	Oxford Foot Model	24.3 (8.7)	4, 6	23 (2.5)
			Flatfoot	9					20.1 (1.3)	2, 7	23.7 (5.7)
Schuh 2013 [7]	28	Double arthrodesis patients with AAFD	Double arthrodesis	14	Patients who underwent double arthrodesis (subtalar and talonavicular) with surgical indications of AAFD stage III	-	X-ray	Plantar loading parameters (emed/D capacitive pressure measurement platform)	65.8 (44–81)	4, 10	29.4 (20.6–41.2)
			Healthy	14					58.4 (47–87)	4, 10	27.7 (22.3–37.8)
* Shin 2019 [24]	78	Flatfoot female	Severe flatfoot	16	(1) clinically diagnosed flatfoot deformity (hindfoot valgus and forefoot abduction); (2) female; (3) over 50 years old; and (4) lateral talus-first metatarsal angle (Meary angle) more than 10 degrees on standing lateral radiograph.	(1) arthritis of more than moderate degree with symptoms associated with the lower extremity joints other than the ankle (hip and ankle joints); (2) neuromuscular involvement of the lower extremities such as cerebral palsy; (3) spinal pathology limiting activities of daily living; (4) other deformities such as tarsal coalition and vertical talus; and (5) any history of surgery involving both lower extremities.	X-ray	DuPont Foot Model	64 (9)	16, 0	28.1 (4.8)
			Moderate flatfoot	20					62.5 (7.3)	20, 0	24.8 (2.9)
			Healthy	42					64 (2.8)	42, 0	24.2 (3.1)
Ellis 2010 [8]	20	LCL with AAFD	Lateral foot pain	10	(1) For flatfoot patients, discomfort level should have a value of ≥5 on a VAS for pain.; (2) for controls, the discomfort level should have a value of <2 on VAS	Pain in the region of the sinus tarsi, the lateral or dorsal aspect of the calcaneocuboid joint, or the dorsal aspect of the anterior part of the calcaneus	X-ray	EMED-ST plantar pressure platform	56.5 (45.8–61.8)	7, 3	-
			No lateral foot pain	10					53 (48.8, 65)	7, 3	-
Petje 1997 [27]	33	Flatfoot	Flatfoot	20	History of Lisfranc subluxation injuries + development of mobile flatfoot deformity	-	X-ray	EMED-SF system	49 (11)	11, 2	-
			Healthy	13					-	-	-
Barske 2013 [18]	26	Stage II AAFD	LCL	13	Undergone unilateral LCL surgery between 2006 and 2009 for stage II AAFD by 1 of 2 fellowship-trained foot and ankle surgeons	Inability to ambulate 50 ft without significant discomfort or difficulty, had a comorbid condition (e.g., insensate feet, metatarsus primus varus) in the same foot as aafd surgery, had a history of ipsilateral lower extremity pain or surgery not due to aafd, or had a postoperative infection or other serious operative complication	X-ray	Force gauge 3D motion analysis (model SML-25 interface)	57.8 (10.3)	12, 1	32.3 (8.9)
			Healthy	13					57.2 (5.4)	10, 3	25.5 (3.8)
Khan 2023 [28]	48	Flatfoot female	Healthy	-	-	Foot deformities other than flatfoot, pregnancy, history of accidents, previous surgeries, or systemic or inflammatory conditions affecting foot joints	X-ray	P-WALK for plantar pressure distribution	20.38 (1.1)	48, 0	-
			FPI positive	-							
			FPI and X-ray positive	-							

Abbreviation. BMI: body mass index; AAFD: adult acquired flatfoot deformity; PTT: posterior tibialis tendon; NSAIDs: non-steroidal anti-inflammatory drugs; PPV: pes planovalgus; MLA: medial longitudinal arch; GRF: ground reaction forces; MRI: magnetic resonance imaging; US: ultrasonography; AOFAS: American Orthopaedic Foot and Ankle Society; LCL: lateral column lengthening; PTTD: posterior tibial tendon dysfunction; VAS: visual analog scale; FPI: Foot Posture Index-6. * Study contributed to the quantitative meta-analysis of spatio-temporal parameters.

**Table 2:** Quality assessment utilizing the Methodological Index for Non-Randomized Studies (MINORS).

Author (Year)	1. A Clearly Stated Aim	2. Inclusion of Consecutive Patients	3. Prospective Collection of Data	4. Endpoints Appropriate to the Aim of the Study	5. Unbiased Assessment of the Study Endpoint	6. Follow-Up Period Appropriate to the Aim of the Study	7. Loss to Follow-Up Less Than 5%	8. Prospective Calculation of the Study Size	9. An Adequate Control Group	10. Contemporary Groups	11. Baseline Equivalence of Groups	12. Adequate Statistical Analyses	Total Score
Comparative Studies													0–24
Petje 1997 [27]	2	1	0	2	1	2	2	0	2	2	1	2	17
Ness 2008 [22]	2	2	0	2	1	0	2	0	2	0	1	2	14
Levinger 2010 [23]	2	0	1	2	1	0	2	0	2	2	1	2	15
Prachgosin 2015 [21]	2	1	2	2	1	0	2	0	2	2	1	2	17
Wang 2019 [20]	2	1	2	2	2	0	2	0	2	1	1	2	17
Shin 2019 [24]	2	1	0	2	1	2	2	0	2	2	1	2	17
Phan 2021 [19]	2	1	2	2	2	0	2	0	2	1	1	2	17
Khan 2023 [28]	2	0	2	2	1	0	2	0	2	2	1	2	16
Barske 2013 [18]	2	0	0	2	1	0	2	0	2	2	1	2	14
Schuh 2013 [7]	2	2	0	2	2	2	2	2	2	2	2	2	22
Ellis 2010 [8]	2	0	0	2	1	2	2	0	2	2	2	2	17
Chong 2015 [15]	2	2	2	2	1	2	2	0	2	2	1	2	20
Non-Comparative Studies													0–16
Colo 2021 [16]	2	2	0	2	1	2	1	0					10
Oeffinger 2000 [14]	2	1	1	2	1	1	0	0					8
Parsons 2010 [17]	2	2	1	2	1	2	2	0					12

**Table 3:** Summary of reported kinematic parameter alterations in flatfoot compared with healthy controls.

Study	Kinematic Analysis Modality	Gait Parameters Consistently Altered in Flatfoot Compared with Healthy Controls	Dynamic Biomarker Potential
Shin 2019 [24]	DuPont Foot Model (15-marker MFM *)	↓ Cadence; ↓ gait velocity; ↓ stride length; ↓ step width; ↓ hindfoot ROM ^†^ (sagittal/transverse); ↓ hallux sagittal ROM; ↑ forefoot abduction and supination	Gait velocity, stride length, cadence, hindfoot sagittal ROM, forefoot abduction
Ness 2008 [22]	Milwaukee Foot Model (radiographic indexing)	↓ Stride length; ↓ cadence; ↓ gait velocity; ↑ hindfoot eversion; ↓ hindfoot dorsiflexion; ↓ forefoot plantarflexion and varus thrust; ↓ hallux dorsiflexion	Hindfoot eversion, hindfoot dorsiflexion, hallux dorsiflexion
Prachgosin 2015 [21]	Oxford Foot Model	↑ MLA ^‡^ eversion moment; ↓ MLA deformation angle; ↑ hindfoot plantarflexion and internal rotation; ↑ forefoot abduction; altered GRF (↓ vertical late stance; ↑ medial mid stance)	MLA eversion moment, MLA deformation angle, GRF vertical and medial
Levinger 2010 [23]	Oxford Foot Model	↑ Forefoot plantarflexion and abduction; ↑ hindfoot internal rotation; ↓ forefoot adduction; trend ↑ hindfoot eversion	forefoot abduction, hindfoot internal rotation
Phan 2021 [19]	Biplanar fluoroscopy	↑ Relative joint surface velocity (tibiotalar; subtalar; calcaneocuboid joints); ↑ tibiotalar inversion/eversion and internal/external rotation ROM; ↑ talonavicular eversion and external rotation	Joint surface velocity, tibiotalar inversion/eversion ROM

* MFM: multisegmental foot model; ^†^ ROM: range of motion; ^‡^ MLA: medial longitudinal arch; GRF: ground reaction forces; ↑ indicates increased; ↓ indicates decreased.

## Data Availability

The datasets generated and/or analyzed during the current research are available from the corresponding author upon reasonable request.

## Supplementary Materials

The following supporting information can be downloaded at: https://www.mdpi.com/article/10.3390/jcm15093324/s1, PRISMA 2020 checklist.
